# Multifunctional alkalophilic α-amylase with diverse raw seaweed degrading activities

**DOI:** 10.1186/s13568-021-01300-x

**Published:** 2021-10-20

**Authors:** Xiaoqian Gu, Liping Fu, Aihong Pan, Yuanyuan Gui, Qian Zhang, Jiang Li

**Affiliations:** 1 Key Lab of Ecological Environment Science and Technology, First Institute of Oceanography, Ministry of Natural Resources, 266061 Qingdao, China; 2 CAS and Shandong Province Key Laboratory of Experimental Marine Biology, Center for Ocean Mega-Science, Institute of Oceanology, Chinese Academy of Sciences, 266071 Qingdao, China; 3 College of Environmental Science and Engineering, Qingdao University, 266071 Qingdao, China; 4 Key Lab of Ecological Environment Science and Technology, The First Institute of Oceanography, SOA, Qingdao, 266061 China

**Keywords:** Metagenomic, α-amylase, Multifunctional enzyme, Enzymatic digestion

## Abstract

Uncultured microbes are an important resource for the discovery of novel enzymes. In this study, an amylase gene (*amy2587*) that codes a protein with 587 amino acids (Amy2587) was obtained from the metagenomic library of macroalgae-associated bacteria. Recombinant Amy2587 was expressed in *Escherichia coli* BL21 (DE3) and was found to simultaneously possess α-amylase, agarase, carrageenase, cellulase, and alginate lyase activities. Moreover, recombinant Amy2587 showed high thermostability and alkali resistance which are important characteristics for industrial application. To investigate the multifunctional mechanism of Amy2587, three motifs (functional domains) in the Amy2587 sequence were deleted to generate three truncated Amy2587 variants. The results showed that, even though these functional domains affected the multiple substrates degrading activity of Amy2587, they did not wholly explain its multifunctional characteristics. To apply the multifunctional activity of Amy2587, three seaweed substrates (*Grateloupia filicina, Chondrus ocellatus*, and *Scagassum*) were digested using Amy2587. After 2 h, 6 h, and 24 h of digestion, 121.2 ± 4 µg/ml, 134.8 ± 6 µg/ml, and 70.3 ± 3.5 µg/ml of reducing sugars were released, respectively. These results show that Amy2587 directly and effectively degraded three kinds of raw seaweeds. This finding provides a theoretical basis for one-step enzymatic digestion of raw seaweeds to obtain seaweed oligosaccharides.

## Introduction

α-Amylases hydrolyze their substrate by breaking the α-1,4-glycosidic bonds in starch molecules releasing glucose, maltose, and oligosaccharide chains as the products (Gupta et al. 2003; Kandra 2003; Rajagopalan and Krishnan 2008). This enzymatic process is one of the earliest to be applied industrially for the production of sugars. α-amylases are also common in animals, plants, and microorganisms (Souza and Magalhães 2010; Yang et al.^2010^; Kandra 2003).

Traditionally, enzymes were believed to have high selectivity or specificity for their substrates and reactions (Dan 2020). However, enzymes with cross-reactivity or promiscuity have been discovered and characterized (Liu et al. 2016; Khersonsky et al. 2006; Pocker and Stone 1965), and increasing numbers of multifunctional enzymes that can hydrolyze various substrates to produce oligosaccharides have been reported.Multifunctional enzymes (also called moonlighting enzymes or promiscuous enzymes) may be a common mechanism of communication and cooperation between different functions and pathways (Carbonell 2011; Jeffery 2003a, b; Jeffery and Constance 2003; Huberts and Klei 2010). Two important characteristics of moonlighting proteins are multiple functions and independency of each function (Huberts and Klei 2010; Cheng et al. 2012). To date, many multifunctional α-amylase have been reported, such as, α-amylase Amy63, possessing agarase, carrageenase activity was reported from marine bacterium *Vibrio alginolyticus* 63 (Liu et al. 2016) and α-amylase Amy19, possessing agarase, carrageenase, and cellulase activity was obtained from Fosmid genomic library of hot spring bacterium *Bacillus* BI-19 (Li et al. 2017).

Multifunctional enzymes are beneficial to living organisms since they expand the biological functions of an organism without the burden of an expanding genome (Jeffery 1999). Moreover, multifunctionality can provide a switch point in biochemical or signaling pathways to enable organisms to better adapt to their environment (Jeffery 2004). However, the multifunctional mechanism was still underexplored. Previously study showed a bifunctional enzyme from the hyperthermophilic bacterium *Caldicellulosiruptor bescii*, has two catalytic domain, which are responsible for different function, respectively (Ye et al. 2012), but most of reported multifunctional enzymes only have one catalytic domain, which can play multifunction simultaneously (Huy et al. 2013; Xue et al. 2015; Yang et al. 2015). To identify the possible functional domain responsible for the multifunctional property of Amy63, three successive conserved domains were deleted one by one, even though the result showed that GH70 homologs might play an important role in the multifunctionality of Amy63, but the exact relationship between catalytic domain and the multifunctionality of Amy63 need to be elucidated further (Liu et al. 2016).

Marine algae have been used extensively in medicine, cosmetics, and food, mostly because of the abundant polysaccharides that they contain. Furthermore, algal oligosaccharides have been found to specifically inhibit viruses (Cáceres et al. 2000; Ji et al. 2010), tumors (Hiroishi et al. 2001), and the coagulation process (Alban et al. 2002). Thus, new methods for obtaining oligosaccharides are of interest in several fields. Complex polysaccharides, such as agar, alginates, and carrageenans, are largely present as cell wall components of seaweeds, where they promote structural integrity and shield the host from pathogens and predators. Traditional methods to prepare oligosaccharides include hydrolysis by acids, oxidation, radiation, microwave, and enzymatic digestion (Duan et al. 2016), but an initial step was required to extract polysaccharide substrates from seaweeds before enzymatic degradation, which can cause high energy consumption. Therefore, considering efficiency and cost, the multifunctional enzyme that can directly and effectively degrade raw seaweeds to obtain seaweed oligosaccharides is very attractive for use in industry and a technologically feasible approach with environmental and economic advantages.

Compare with screening the activities of proteins isolated from culturable microbes, uncultured microbes have long been considered as an important resource for the discovery of novel enzyme. Recently, the application of metagenomic technologies to explore marine environments has provided a new way to screen for novel enzymes, especially those produced by uncultured microbes (Tian et al. 2012; Bhattacharyya et al. 2014). In this study, we report a novel multifunctional α-amylase (Amy2587) with agarase, carrageenase, cellulase, and alginate lyase activities that we discovered by screening a macroalgae-associated bacteria metagenomic library. The purified Amy2587 was characterized and its multifunctional mechanism was explored by deleting three motifs in its sequence to obtain truncated proteins. The study will provide a potential method for one-step enzymatic digestion to prepare seaweed oligosaccharides directly from raw seaweed.

## Materials and methods

### Construction of the metagenomic library

Macroalgae samples (*Grateloupia filicina, Chondrus ocellatus*, and *Scagassum*) were collected from Halmahera Island (0^o^ 36′N, 127^o^ 52′E), Indonesia. The samples were washed with sterilized seawater, then cut into pieces and placed in a sterile tube. Sterile water was added and shaken three times with a vortex oscillator for 2 min each time. Then, sterile filter membranes (50 mm, 0.22-µm pore size) were used to collect the bacteria for later use. A FastDNA Spin Kit for Soil (MP Bio) was used to extract the genomic DNA from the collected bacteria samples. After testing, the qualified genomic DNA was sent to Jingneng Biotechnology Co., Ltd. (Shanghai, China) for sequencing, assembly, and functional annotation.

### Plasmids, vectors, and substrates

Plasmid pET-30a (+) and *E. coli* BL21 (DE3) were purchased from Tiangen Biotech Co., Ltd. (Beijing, China). Soluble starch, agarose, carrageen, sodium cellulose, and alginate were purchased from Sinopharm Group Chemical Reagent Co., Ltd. (Shanghai, china). *Grateloupia filicina*, *Chondrus ocellatus*, and *Scagassum* were collected from Yangkou Beach in Qingdao, China.

### Gene synthesis and sequence analysis of Amy2587

The metagenomic data of the macroalgae-associated bacteria samples were analyzed and the *amy2587* gene sequence was obtained by screening the metagenomic library and used as the template to synthesize the target gene. The gene was synthetized by Nanjing Kingsley Biotechnology Co., Ltd. (Nanjing, China). BLAST Search (http://blast.ncbi.nlm.nih.gov/Blast.cgi) were used to identify the *amy2587* sequence and the DNAMAN software package (http://www.lynnon.com/) was used for multiple sequence alignment. And the motifs were analyzed using Motif Search (http://www.genome.jp/tools/motif/).

### Expression of *amy2587* and purification of Amy2587

The *amy2587* gene and pET-30(a) were digested by *Bam*HI and *Xba*I endonucleases respectively, then ligated by T4 DNA ligase to construct the recombinant plasmid Amy2587+pET-30(a). The recombinant Amy2587 was expressed in *E. coli* BL21 (DE3). The obtained transformants were incubated on LB medium (50 µg/ml kanamycin) with constant shaking at 150 rpm at 37 °C. The Isopropyl-β-d-thiogalactopyranoside (IPTG) concentration (0 mmol/L, 0.1 mmol/L, 0.3 mmol/L, 0.5 mmol/L, 0.7 mmol/L, 1.0 mmol/L) of Amy2587 was optimized, and the enzyme activity of Amy2587 was determined by DNS method, so as to determine the optimal IPTG concentration of Amy2587. IPTG was added to express the fusion protein when the OD_600_ reached 0.6. After induction for 16 h at 16 °C, the cells were collected, placed on ice, and crushed using an ultrasonic cell crushing apparatus.

An Ni-NTA His Tag Kit (Novagen) was used to purify the recombinant Amy2587. First, binding buffer (10 mM imidazole, 50 mM NaH_2_PO_4_, 300 mM NaCl, pH 8.0) was used to wash the recombinant Amy2587, then elution buffer with different concentrations of imidazole (20 mM, 80 mM, 140 mM, and 200 mM) was used to elute the recombinant Amy2587 (Riera et al. 2003). Finally, the target protein Amy2587 was assessed by sodium dodecyl sulphate–polyacrylamide gel electrophoresis (SDS–PAGE) (Blakesley and Boezi 1977).

### Substrate specificity of Amy2587

To determine the multifunctionality of Amy2587, we studied its substrate specificity. 100 µL purified Amy2587 (0.2 mg/mL) and 900 µL substrate (0.1% soluble starch, 0.1% agarose, 0.1% carrageen, 0.1% sodium cellulose, and 0.1% alginate) were incubated for 40 min at 50 °C, and the Amy2587 activity was measured by the 3,5-dinitrosalicylic acid (DNS) method (Chi et al. 2014). One unit of enzyme activity was defined as the amount of enzyme that can catalyze the release of 1 µmol of reducing sugar per minute.

### Characterization of Amy2587

To determine the effect of pH on Amy2587 activity, 100 µL purified Amy2587 (0.2 mg/mL) and 900 µl substrate (0.1% soluble starch, 0.1% agarose, 0.1% carrageen, 0.1% sodium cellulose, and 0.1 % alginate) were incubated in different buffer systems from pH 4.0–11.0 (pH 4.0−7.0, Na_2_HPO_4_-citric acid; pH 7.1−8.9, Tris-HCl; pH 9.0−10.6, glycine-NaOH) for 40 min at 50 °C. Amy2587 activity was determined by the DNS method. The highest detected enzyme activity was defined as 100 %.

To determine the effect of temperature on Amy2587 activity, 100 µL purified Amy2587 (0.2 mg/mL) and 900 µL substrate (0.1 % soluble starch, 0.1% agarose, 0.1% carrageen, 0.1% sodium cellulose, and 0.1% alginate) were incubated at 10 °C, 20 °C, 30 °C, 40 °C, 50 °C, 60 °C, 70 °C, and 80 °C for 40 min. The highest detected enzyme activity was defined as 100%.

To determine the thermostability of Amy2587, 100 µL purified Amy2587 (0.2 mg/mL), which had been pre-incubated at 40 °C, 50 °C, and 60 °C for 0 to 24 h, and 900 µl substrate (0.1% soluble starch, 0.1 % agarose, 0.1 % carrageen, 0.1% sodium cellulose, and 0.1% alginate) were incubated for 40 min. The highest detected enzyme activity was defined as 100%.

To determine the effect of metal ions (2 mM) on Amy2587, metal cations (Sr^2+^, Ni^2+^, Ca^2+^, Ba^2+^, Mn^2+^, Mg^2+^, Fe^2+^, Fe^3+^, K^+^, Cu^2+^, Na^+^) were added to the corresponding reaction mixtures and incubated for 40 min at 50 °C and pH 7.0, the standard assay conditions. The enzyme activity in the absence of metal ions was defined as 100%.

### Kinetic parameters assay

The concentrations of the five substrates in the assay system were changed, and changes in the enzymatic reaction rates were measured under the standard assay conditions. The Lineweaver-Burk double-reciprocal method (Morrison 2002) was used to obtain the kinetic parameters and determine the kinetic behaviors of Amy2587.

#### Variant assay of Amy2587

**Fig. 1:**
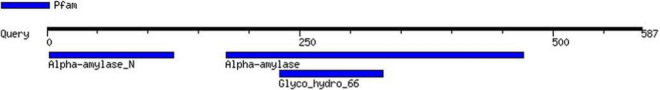
Sequence analysis of the α-amylase Amy2587. The Pfam motifs are indicates by blue line

In order to disclose the possible functional domain responsible for the multifunctional property of Amy2587, the potential three functional domains (α-amylase_N, α-amylase, and Glyco_hydro_66) (Fig. 1) were found by motif search. Then the truncated genes without these functional domain, named *amy2587a* (1428 bp), *amy2587b* (918 bp), and *amy2587c* (1491 bp), were synthetized by Nanjing Kingsley Biotechnology Co., Ltd. (Nanjing, China). Truncates were constructed and heterologously expressed in *E. coli* BL21 cells.

#### Raw seaweeds digestion using Amy2587

We employed one-step enzymatic digestion method to obtain oligosaccharides directly from raw seaweed using the novel multifunctional α-amylase Amy2587 as follows. Purified enzyme solution (0.2 mg/mL) was added to artificial seawater containing 2 % (w/v) of dried red seaweeds, *Grateloupia filicina*, which mainly produces carrageenan, and *Chondrus ocellatus*, which mainly produces agar, and dried brown seaweed, *Scagassum*, which mainly produces alginate. The reaction mixtures were incubated for 0.25 h, 0.5 h, 1 h, 2 h, 6 h, 12 h, and 24 h at 50 °C under the constant shaking at 150 rpm. The ability of Amy2587 to digest the three raw seaweed substrates was demonstrated by measuring the reducing sugar content using the 3,5-dinitrosalicylic acid (DNS) method (Chi et al. 2014).

## GenBank accession number

The complete open reading frame of Amy2587 has been deposited in the GenBank database under accession number MW839461.1.

## Results

### Sequence analysis of Amy2587

**Fig. 2:**
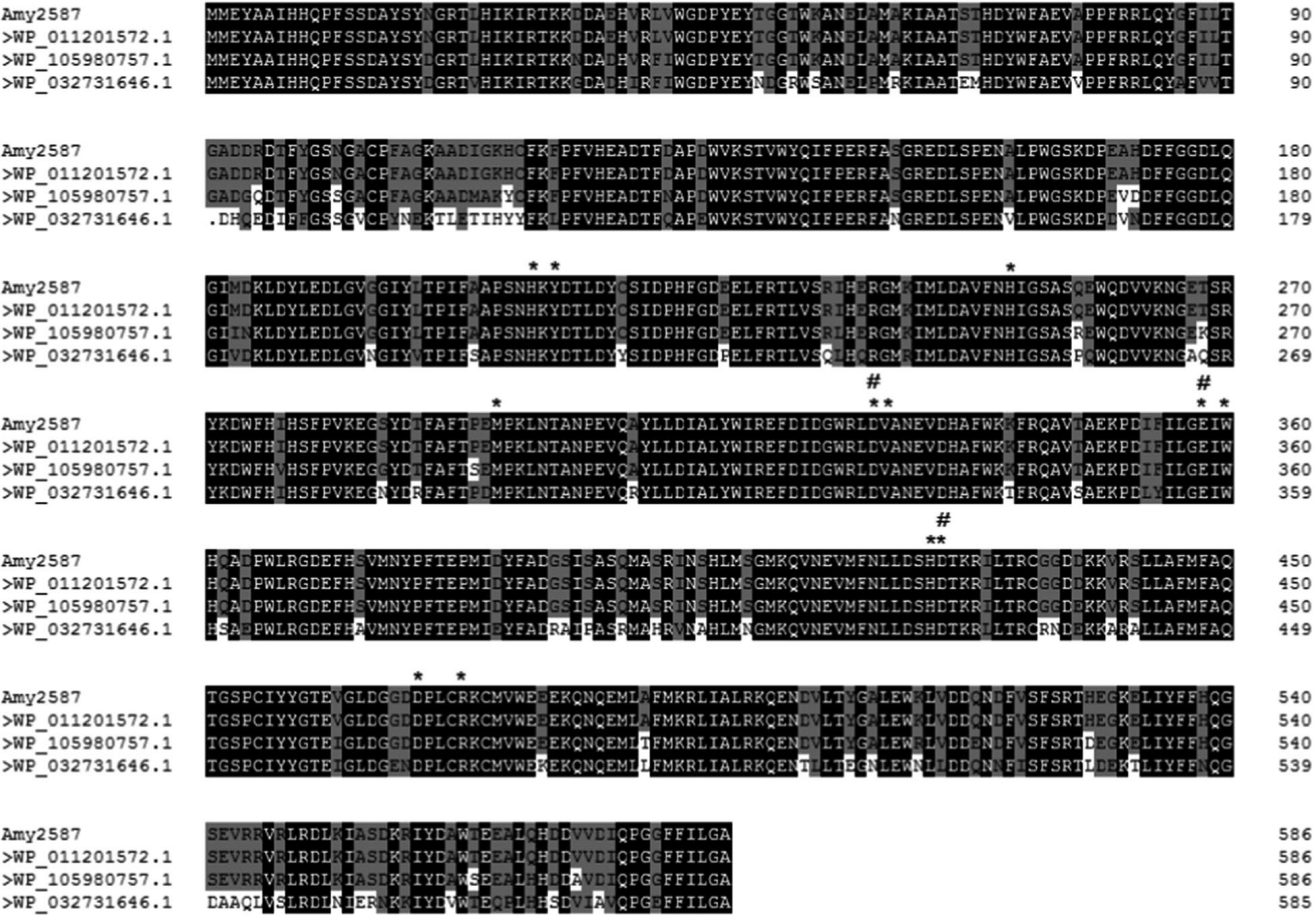
Multiple sequence comparison of the α-amylase Amy2587

A novel amylase gene *amy2587* was screened from the metagenomic analysis of macroalgae-associated bacteria. The *amy2587* gene was 1785-bp long and coded a 587-amino acid long protein with a theoretical molecular weight of 67.46 kDa. Sequence analysis (Fig. 1) showed that the encoded protein had three Pfam motifs: α-amylase_N, α-amylase, and Glyco_hydro_66. And the Amy2587 protein sequences were compared with those of the reported amylase (Fig. 2): WP_011201572.1, WP_105980757.1 and WP_032731646.1 showed that the deduced amino acid sequence of *amy2587* had high similarity (78–100 %). Based on multiple sequence comparison, Amy2587 had 12 active sites: His209-Tyr211-His251-Met296-Asp329-Val330-Glu358-Trp360-His424-Asp425-Asp469-Arg473 and 3 catalytic sites: Asp329-Glu358-Asp425.

### Expression of *amy2587* and purification of Amy2587

**Fig. 3:**
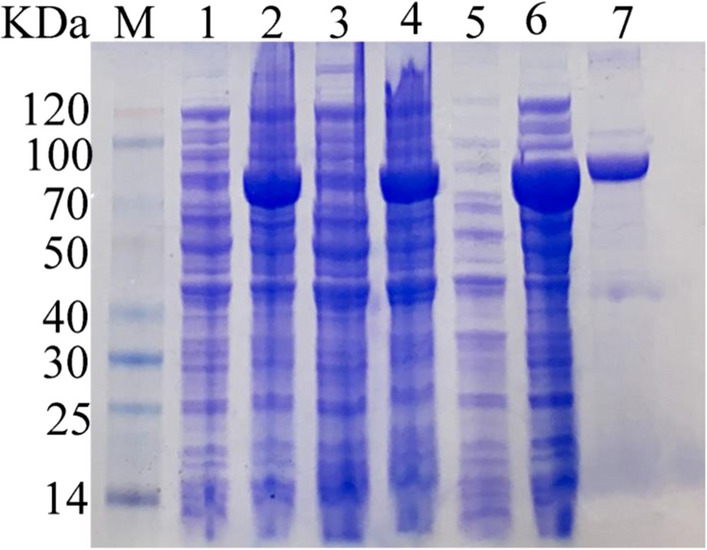
SDS–PAGE analysis of the target protein Amy2587. M: Protein marker; Lane 1: Whole-cell of recombinant *Escherichia coli* BL21 (DE3) cells harboring pET-30(a)-*amy2587* before induction; Lane 2: Whole-cell of recombinant *Escherichia coli* BL21 (DE3) cells harboring pET-30(a)-*amy2587* after induction; Lane 3: Supernatant of recombinant *Escherichia coli* BL21 (DE3) cells harboring pET-30(a)-*amy2587* before induction; Lane 4: Supernatant of recombinant *Escherichia coli* BL21 (DE3) cells harboring pET-30(a)-*amy2587* after induction; Lane 5: Precipitate of recombinant *Escherichia coli* BL21 (DE3) cells harboring pET-30(a)-*amy2587* before induction; Lane 6: Precipitate of recombinant *Escherichia coli* BL21 (DE3) cells harboring pET-30(a)-amy2587 after induction; Lane 7: Purified Amy2587

We optimized the IPTG concentration (0 mmol/L, 0.1 mmol/L, 0.3 mmol/L, 0.5 mmol/L, 0.7 mmol/L, 1.0 mmol/L) for protein expression and found that 0.5 mM was optimum level determined by DNS method. The SDS–PAGE analysis (Fig. 3) showed that a single target band of protein (approximate 68 kDa) was purified when the concentration of the target protein in the imidazole eluent was 80 mM. Compared with Amy2587 without induction, the expression level of Amy2587 with IPTG induction (0.5 mM) was significantly increased from the SDS-PAGE result (Fig. 3), which confirmed the correct expression of *amy2587* in *E. coli* BL21 cells.

### Multifunctionality of recombinant Amy2587

The purified Amy2587 exhibited specific activity levels of 63.38 ± 0.02 U/mg, 18.37 ± 0.04 U/mg, 18.22 ± 0.02 U/mg, 14.51 ± 0.03 U/mg, and 17.74 ± 0.03 U/mg toward five substrates (soluble starch, agar, carrageen, sodium cellulose, and alginate) (Table 1). These results confirmed that Amy2587 was a multifunctional α-amylase that possessed amylase, agarase, carrageenase, cellulase, and alginate lyase activities simultaneously.

**Table 1:** Specific activity levels (U/mg) of Amy2587 and three truncated Amy2587 variants with different substrates

Substrates	Amy2587	Amy2587a	Amy2587b	Amy2587c
Soluble starch Agar Carrageen Sodium cellulose Alginate	63.38 ± 0.02 18.38 ± 0.04 18.22 ± 0.02 14.51 ± 0.03 17.44 ± 0.03	44.30 ± 0.03 5.86 ± 0.02 9.09 ± 0.02 8.68 ± 0.05 9.87 ± 0.03	37.43 ± 0.03 5.82 ± 0.03 5.17 ± 0.02 9.79 ± 0.01 5.73 ± 0.02	30.26 ± 0.01 9.16 ± 0.01 6.76 ± 0.03 7.88 ± 0.02 9.89 ± 0.02

The truncated Amy2587 variants, Amy2587a, Amy2587b, and Amy2587c have had the α-amylase_N motif, α-amylase, and Glyco_hydro_66 motifs deleted, respectively

### Biochemical characterization of recombinant Amy2587

**Fig. 4:**
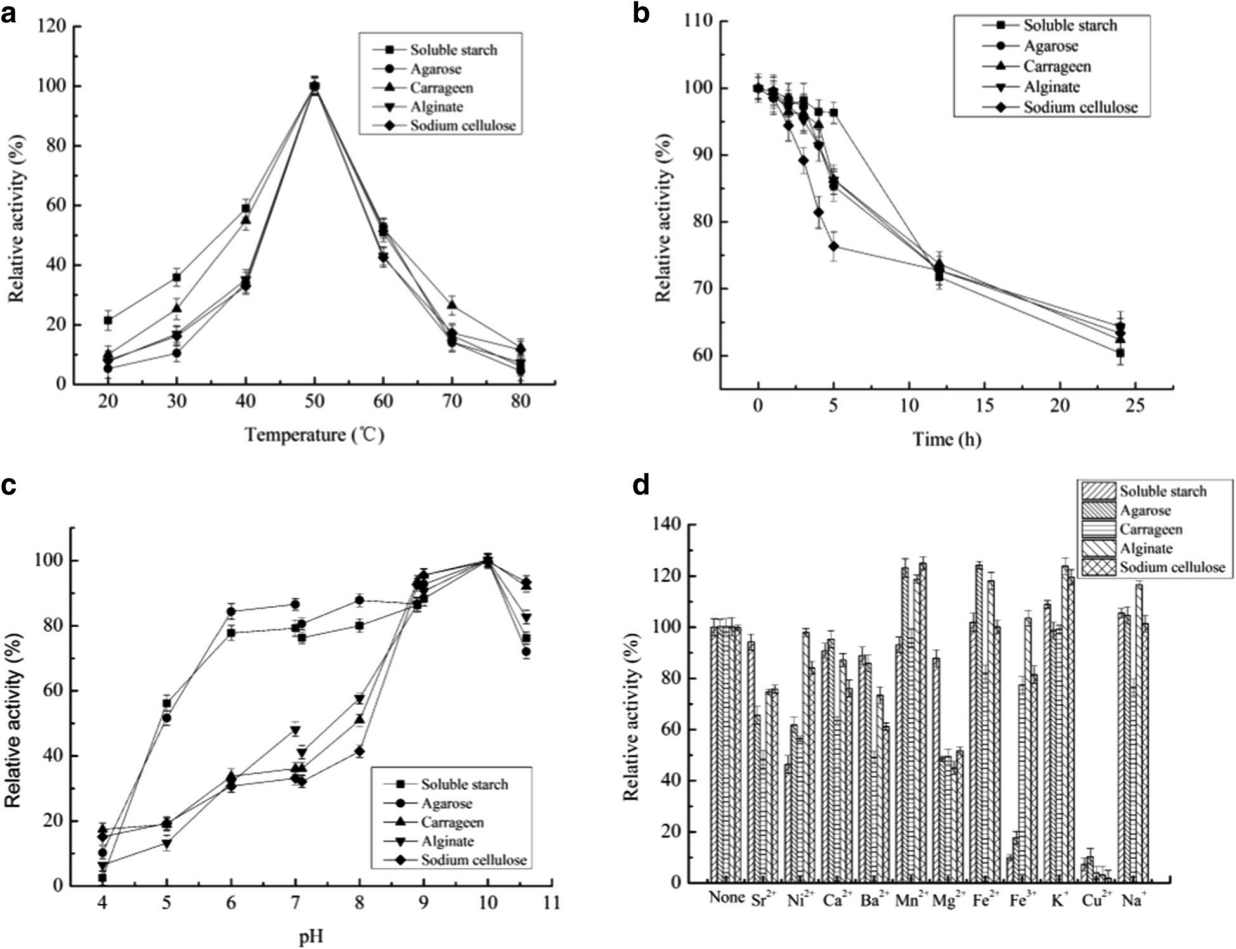
Effects of temperature, pH and metal ions on the activity of Amy2587 with different substrates. **a** Effects of temperature on the activity of Amy2587 with different substrates; **b** Thermostability of Amy2587 at different temperatures and time points with different substrates; **c **Effects of pH on the activity of Amy2587 with different substrates; **d** Effects of metal ions and metal salts on the activity of Amy2587 with different substrates

The highest activities for the α-amylase, agarase, carrageenase, cellulase, and alginate lyase were obtained at 50 °C, with about 50 % initial activity retained at 40–60 °C (Fig. 4a). Notably, Amy2587 showed high thermostability with almost 85 % of its original activity retained after incubation at 50 °C for 4 h. With increasing incubation times, the enzyme activity gradually decreased, but was still more that 60 % after 24 h of incubation (Fig. 4b).

The highest activities for the α-amylase, agarase, carrageenase, cellulase, and alginate lyase were obtained at pH 10.0 (Fig. 4c). Notably, Amy2587 showed high multifunctional activity, with almost 70% of its original activity retained after incubation at pH 11.0 for 24 h. The results indicated that Amy2587 was an alkaliphile enzyme, and such enzymes play important roles in industrial biotransformations.

The metal ions had different effects on Amy2587 activity (Fig. 4d). Mn^2+^, Fe^2+^, K^+^, and Na^+^ had different degrees of promoting effects, and Sr^2+^, Ni^2+^, Ca^2+^, Ba^2+^, Mg^2+^, and Fe^3+^ had different degrees of inhibiting effects on Amy2587 activity. Notably, Cu^2+^ dramatically reduced Amy2587 activity, and all the multifunctional enzyme activities were almost completely lost.

### Kinetic parameters of Amy2587

**Fig. 5:**
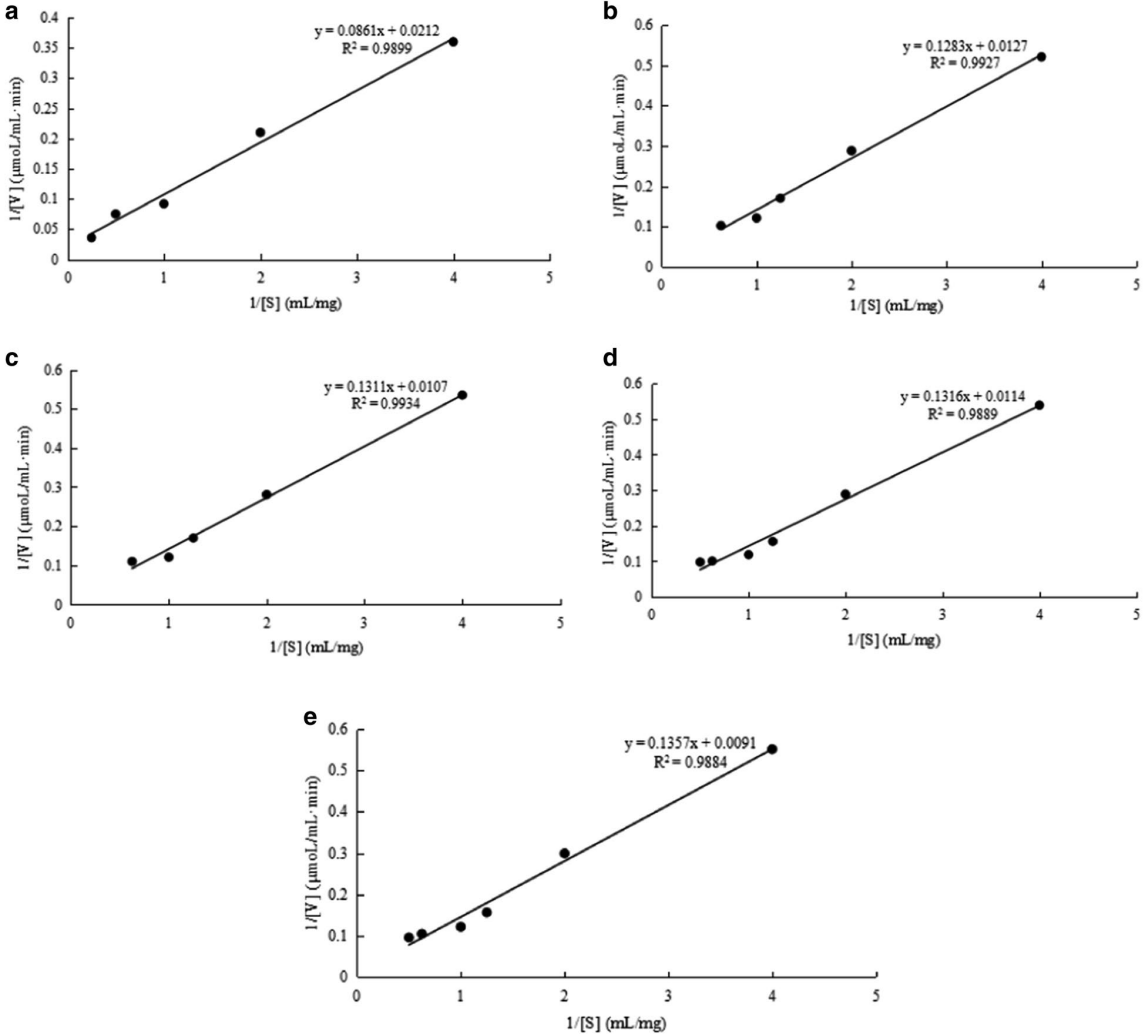
Kinetic parameters of Amy2587 with different substrates. **a** Amylase activity; **b** Agarase activity; **c** Carrageenase activity; **d** Cellulase activity; **e** Alginate lyase activity

The *K*_m_ values of Amy2587 for the five substrates, soluble starch, agarose, carrageen, sodium cellulose, and alginate, were 4.06 ± 0.04 mg/mL, 10.10 ± 0.03 mg/mL, 12.25 ± 0.04 mg/mL, 11.54 ± 0.06 mg/mL and 14.91 ± 0.06 mg/mL, respectively. The *V*_max_ values of Amy2587 for the five substrates, soluble starch, agarose, carrageen, sodium cellulose, and alginate, were 47.16 µmol/mL·min, 78.74 µmol/mL·min, 93.46 µmol/mL·min, 87.71 µmol/mL·min and 109.89 µmol/mL·min, respectively (Fig. 5).

### Variant assay of Amy2587

To further understand the multifunctionality of Amy2587, three functional domains coded in the *amy2587* sequence were knocked out. The truncated Amy2587 variants *amy2587a* (1428 bp), *amy2587b* (918 bp), and *amy2587c* (1491 bp) were successfully expressed and purified. The enzymatic activity of Amy2587 was higher than that of the three truncated Amy2587 variants (Amy2587a, Amy2587b, and Amy2587c) for all five substrates, soluble starch, agarose, carrageen, sodium cellulose, and alginate (Table 1).

### Digestion of raw seaweeds by Amy2587

**Fig. 6:**
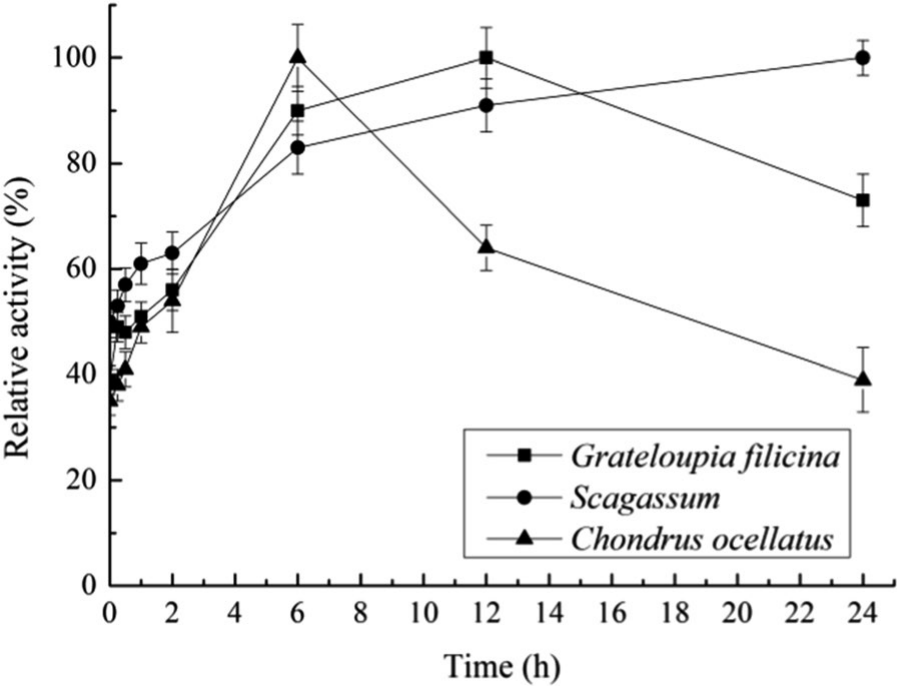
Relative activity of Amy2587 during degradation of three seaweed substrates, *Grateloupia filicina*, *Chondrus ocellatus*, and *Scagassum*

**Fig. 7:**
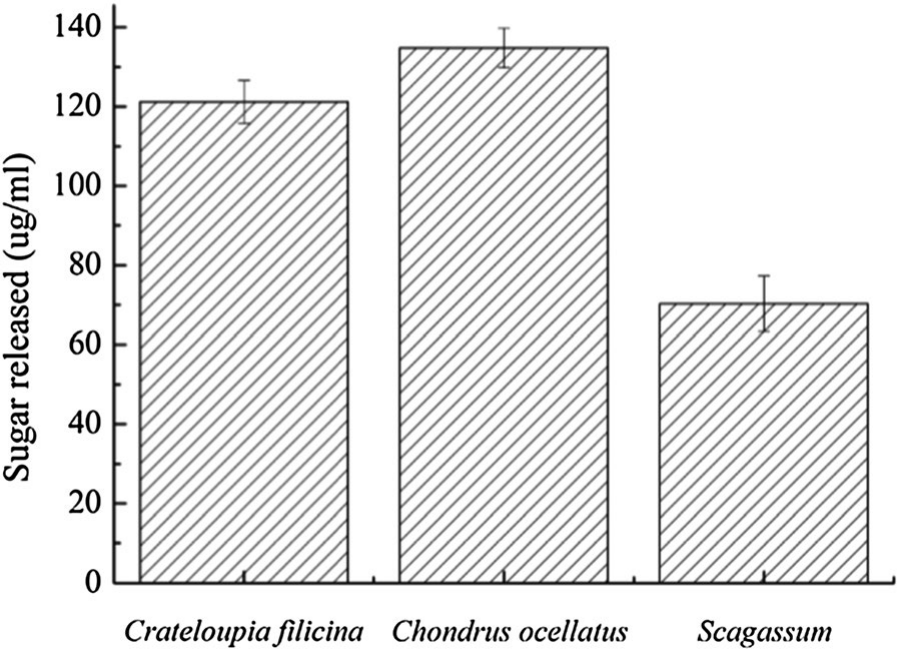
Reducing sugars released during Amy2587 degradation of three seaweed substrates, *Grateloupia filicina*, *Chondrus ocellatus*, and *Scagassum*, after 12 h, 6 h, and 24 h, respectively

Amy2587 showed high ability to digest raw seaweeds after incubating for 0.25 h, 0.5 h, 1 h, 2 h, 6 h, 12 h, and 24 h under the standard assay conditions (Fig. 6). During the degradation of the seaweed substrates, *Grateloupia filicina*, *Chondrus ocellatus*, and *Scagassum*, Amy2587 released 121.2± 4 µg/ml, 134.8± 6 µg/ml, and 70.3± 3.5 µg/ml of reducing sugars after 12 h, 6 h, and 24 h, respectively (Fig. 7).

## Discussion

Active screening of culturable microorganisms is still the main method used to obtain polysaccharide-degrading enzymes (Marjolaine et al. 2015). However, the recent explosion of metagenomic data has provided a new way of screening for polysaccharide-degrading enzymes, especially novel enzymes produced by unculturable microorganisms. In this study, an amylase gene *amy2587* was screened from metagenomic library from macroalgae-associated bacteria.

Recombinant Amy2587 was expressed in *E. coli* BL21 (DE3) and characterized. To determine the multifunctionality of Amy2587, we studied its substrate specificity and found that it exhibited strong α-amylase, agarase, carrageenase, cellulase, and alginate lyase activities, simultaneously. Other amylases have been reported to have multifunctional functions. For example, the multifunctional amylase Amy440 produced by *Aquimarina agarilytica* ZC1 was reported to show amylase and agarase activities (Lin et al. 2017); the multifunctional amylase Amy63 produced by *Vibrio alginolyticus* 63 was reported to show amylase, agarase and carrageenase activities (Liu et al. 2016); and the multifunctional amylase Amy19 produced by *Bacillus* BI-19 was reported to show agarase, carrageenase, and cellulase activities (Li et al. 2019). To the best of our knowledge, Amy2587 is the first multifunctional enzyme found to have five enzyme activities at the same time. Considering efficiency and cost, enzymes that exhibit amylase, agarase, carrageenase, cellulase, and alginate lyase activities would have wide industrial applications (Chai et al. 2020).

Amy2587 showed high thermostability and was alkalophilic, which are important characteristics for enzymes that play important roles in biotransformations and industrial production because polysaccharide substrates are colloidal and viscous at low temperatures or high concentrations, which impedes the efficiency of binding between enzymes and substrates (Li et al. 2019).

The effects of the metal ions on Amy2587 activity were not identical. In particular, Cu^2+^ dramatically reduced Amy2587 activity, and all the multifunctional activities were almost lost. Similar results for the effect of Cu^2+^ have been reported for other enzymes, including Amy19 (Li et al. 2019) in the GH70 family and AgaXa (Xie et al. 2013) in the GH118 family. Cu^2+^ decreases enzyme activity by binding with the thiol group in the active site of enzymes (Murashima et al. 2002).

The *K*_m_ value of Amy2587 with soluble starch was similar to the *K*_m_ value for the amylase from *Thermus filiformis* 0rk A2 (5.0 mg/ml) (Egas et al. 1998), and lower than the *K*_m_ values for the amylases from *Bacillus alcalophilus* JN21 (9.64 mg/mL) (Yang et al. 2011) and *Thermococcus* HJ21(45.0 mg/mL) (Wang et al. 2008). The *K*_m_ value of Amy2587 with agarose was similar to the *K*_m_ value for the agarase obtained from *Catenovulum* sp. X3 (10.50 mg/mL) (Xie et al. 2013), and lower than the *K*_m_ values for the agarases from *Agarivorans albus* OAY02 (15.38 mg/mL) (Yang et al. 2014) and *Saccharophagus degradans* 2-40 (41.90 mg/mL) (Dong et al. 2016). The *K*_m_ value of Amy2587 with carrageen was higher than the *K*_m_ values for carrageenases from *Vibrio*. sp. (2.54 mg/mL) (Zhu et al. 2016) and *Pseudoalteromonas* sp. (9.80 mg/ml) (Ma et al. 2010), and lower than the *K*_m_ value for the carrageenase from *Shewanella* sp. Kz7 (716.8 mg/ml) (Wang et al. 2015). The *K*_m_ value of Amy2587 with sodium cellulose was higher than the *K*_m_ values for the cellulases from *Bacillus subtilis* SU40 (1.97 mg/mL) (Asha et al. 2014) and *Bacillus subtilis* I15 (3,59 mg/mL) (Yang et al. 2010a, b). The *K*_m_ value of Amy2587 with alginate was higher than the *K*_m_ value for the alginate lyase from *Vibrio furnissii* H1 (2.28 mg/mL) (Zhu et al. 2018). These results show that Amy2587 had good affinity for all five substrates.

To further understand the multifunctionality of Amy2587, three motifs, Alpha-amylase_N, Alpha-amylase, or Glyco_hydro_66, in the Amy2587 sequence were deleted. The results showed that these functional domains affected the multiple substrates degrading activity of Amy2587, but the multifunctional characteristics were not fully explained. The Amy2587 activity decreased to different degrees in the truncated enzymes, but the amylase activity was not lost and, although the agarase, carrageenase, cellulase, and alginate lyase activities were affected, the multifunctional activities of Amy2587 remained.

The gene sharing model is often used to explain why an enzyme maintains promiscuity, where a gene with a given function is recruited for a different, moonlighting function without any changes in the coding region (Piatigorsky et al. 1989). Furthermore, evolution of the active sites of existing enzymes that promiscuously bind the substrates can allow new enzymatic capabilities to be generated (Khersonsky et al. 2006). Amy2587 originate from metagenomic library of associated-bacteria of macroalgae surface, which were collected from coastal zone near hot springs of Ambon island, Indonesia. Usually, such environment has high temperature but low nutrient content. Liu et al. (2016) hypothesized that these marine bacteria have evolved their existing amylase to allow usage of other carbohydrates like agar and carrageenan as the carbon source when they must survive in a harsh environment without enough starch.

To apply the multifunctional activity of Amy2587, three seaweed substrates (*Grateloupia filicina, Chondrus ocellatus and Scagassum*) were digested using Amy2587. The amount of reducing sugars released by Amy2587 digestion was much higher than the amount released by *Microbulbifer* strain CMC-5 digestion of red seaweed for 10 days (60 µg/mL) (Jonnadula et al. 2009) and higher than the amount released by *Bacillus* sp. SYR4 digestion of seaweed for 120 h (24 µg/mL) (Kang and Kim 2015), but lower than the amount released by bacteria DM1, DM5, and DM15 utilization of *Sargassum* after 72 h (503.3 ± 17.5 µg/mL, 491.6 ± 20 µg/mL, and 376.6 ± 16 µg/mL respectively). These results suggest that the novel multifunctional α-amylase Amy2587 has high potential for seaweed degradation. To our knowledge, this is the first report of an enzyme, not bacteria, that can directly and effectively degrade different kinds of raw seaweeds to obtain seaweed oligosaccharides. Therefore, eco-friendly degradation for the production of oligosaccharides directly from raw seaweed may be possible using Amy2587. This finding provides a theoretical basis for one-step enzymatic digestion of raw seaweeds.

## Data Availability

The complete open reading frame of Amy2587 has been deposited in the GenBank database under accession number MW839461.1.
